# Protective effects of hydrogen sulfide on chronic kidney disease by reducing oxidative stress, inflammation and apoptosis

**DOI:** 10.17179/excli2017-711

**Published:** 2018-01-02

**Authors:** Hassan Askari, Behjat Seifi, Mehri Kadkhodaee, Nima Sanadgol, Mohammed Elshiekh, Mina Ranjbaran, Parisa Ahghari

**Affiliations:** 1 Department of Physiology, Faculty of Medicine, Tehran University of Medical Sciences, Tehran, Iran; 2 Department of Biology, Faculty of Sciences, University of Zabol, Zabol, Iran; 3 Young Researchers and Elite Club, Zahedan Branch, Islamic Azad University, Zahedan, Iran; 4 Department of Physiology, Faculty of Medicine, University of Dongola, Dongola, Sudan; 5 Department of Physiology, Faculty of Medicine, Hamedan University of Medical Sciences, Hamedan, Iran

**Keywords:** chronic kidney disease, hydrogen sulfide, oxidative stress, apoptosis, inflammation, creatinine clearance, blood pressure

## Abstract

The current study aimed to examine the renoprotective effects of long-term treatment with sodium hydrosulfide (NaHS), a prominent hydrogen sulfide donor, in 5/6 nephrectomy animal model. Twenty-four rats were randomly divided into 3 groups including sham-operated group (Sham), 5/6-nephrectomized group (5/6 Nx), and NaHS-treated group (5/6Nx+NaHS). NaHS (30 micromol/l) was added twice daily into the drinking water and renal failure was induced by 5/6 nephrectomy. Twelve weeks after surgical procedure, blood pressure, creatinine clearance (CCr), urine concentration of neutrophil gelatinase associated lipocalin (NGAL) and tissue concentration of malondialdehyde (MDA), superoxide dismutase (SOD), as well as renal morphological changes, apoptosis (cleaved caspase-3) and inflammation (p-NF-κB) were measured. Five-sixth nephrectomy induced severe renal damage as indicated by renal dysfunction, hypertension and significant histopathological injury which were associated with increased NGAL and MDA levels, oxidant/antioxidant imbalance, decreased SOD activity and CCr and also overexpression of p-NF-κB and cleaved caspase-3 proteins. Instead, NaHS treatment attenuated renal dysfunction through reduction of NGAL concentration, hypertension, CCr, oxidant/antioxidant imbalance, inflammation and apoptosis. These findings suggest that long term NaHS treatment can be useful in preventing the progression of CKD by improving oxidant/antioxidant balance and reducing inflammation and apoptosis in the kidney.

## Introduction

Chronic kidney disease (CKD) is a global public health problem (Askari et al., 2016[4]). CKD is defined as decreased estimated glomerular filtration rate (eGFR) lower than 60 mL/min per 1·73 m^2^, or the presence of markers of renal damage in urine such as urinary neutrophil gelatinase-associated lipocalin (uNGAL) (Marcuccilli and Chonchol, 2016[28]; Targher et al., 2014[35]). After initial injury to the kidneys due to systemic hypertension, inflammation and oxidative stress, a compensatory and adaptive response in the nephrons begins which ultimately induces detrimental effects to the renal tissue and causes further nephron loss and end-stage kidney disease. In this context, structural modifications in CKD patients may cause oxidative stress related to an imbalance between free radical's production and antioxidant capacity. This, in turn directly triggers systemic inflammation by activation of redox-sensitive proinflammatory transcription factors and signal transduction pathways which cause apoptosis and further progression of renal injury (Akchurin and Kaskel, 2015[2]; Vaziri, 2008[36]). Currently, the therapeutic options available for managing CKD are not quite efficient and therefore, there lies a subsequent need for effective drug therapies that can prevent progressive damage to the kidneys. Recent evidence suggests that hydrogen sulfide (H_2_S), a novel gaseous signaling molecule similar to nitric oxide (NO) and carbon monoxide (CO), can exhibit renoprotective effects in the animal model of kidney injury (Shui et al., 2016[33]; Wesseling et al., 2015[37]) via antihypertensive, antioxidative, antiapoptotic and anti-inflammatory mechanisms (Ju et al., 2013[24]; Snijder et al., 2014[34]; Wu et al., 2017[38]). Therefore, given the critical role of oxidative stress, hypertension, inflammation and apoptosis in the progression of renal disease and the potent protective actions of H_2_S in kidney disease, the present study was designed to investigate the effect of pharmacological long-term sodium hydrosulfide (NaHS) treatment on 5/6 nephrectomy animal model. 

## Methods

### Experimental design

The experiment procedures were approved by Tehran University of Medical Sciences ethical committee and were in accordance with the National Institutes of Health Publication, Animal Care and Use of Laboratory Animals guidelines. Male Wistar rats, weighing 200-250 g, were housed under standard conditions (12 h light-dark cycle; 20-22 °C) with free access to water and a standard rat diet and randomly assigned to control (n=8) and CKD (n=16) subjects. CKD was induced by a two-step 5/6 nephrectomy (5/6 Nx) as described previously (Askari et al., 2016[4]). All these CKD rats were housed for four weeks to establish the 5/6 nephrectomy model and then equally randomized to receive NaHS (30 micromol/l twice daily) in drinking water or drinking water alone daily for 8 weeks. Sham surgery was performed in the same way as described for CKD with the exception of no manipulation of the kidneys. 12 weeks' post-surgery the values of systolic blood pressure were measured between 13:00 and 16:00 hours by the tail-cuff method connected to a pulse transducer device (MLT125/R; AD Instruments, Castle Hill, NSW, Australia). The transducer was connected to a PowerLab/4SP data-acquisition system (Chart, version 5; AD Instruments). Five measurements were taken for each rat and subsequently averaged. After 12 weeks, the animals were weighed and placed in metabolic cages for a 24 h urine collection and then anaesthetized to obtain their blood samples from the inferior vena cava. Finally, kidneys tissue was taken for oxidative stress status. Part of the kidney was fixed in 10 % formalin for histological assessment and immunohistochemistry.

### Biochemical assay

Urine samples were collected at the end of the experiments and the total volumes were recorded. In conjunction with serum creatinine concentration and urine volume, urinary creatinine concentration was measured by colorimetric methods to calculate creatinine clearance (CCr) using standard formulas. Moreover, urine NGAL levels were measured by lipocalin-2 (NGAL) Rat ELISA Kit (ab119602) according to manufacturer's protocol. 

### Evaluation of renal markers of oxidative stress 

#### Lipid peroxidation (MDA) assay

Malondialdehyde (MDA) levels were measured in tissue samples using the thiobarbituric acid reactive substances (TBARS) method according to the methods of Esterbauer and Cheeseman (Elshiekh et al., 2017[14]; Esterbauer and Cheeseman, 1990[16]). The values were expressed as µmol/100 mg tissue.

#### Superoxide dismutase activity assay

Renal tissue SOD activity was assessed according to the spectrophotometric method of Paoletti and Mocali (1988[29]). Oxidation of NADPH is linked to the availability of superoxide anions in the medium. The values were expressed as U/g tissue.

#### Immunohistochemistry evaluation

Kidney samples of the different groups were fixed in formalin and embedded in paraffin and sections of 3 μm were cut with a microtome and stained with hematoxylin and eosin according to standard procedures. At least 8 random non-overlapping areas at 100 magnifications were observed for the presence of tubular necrosis, tubular dilatations and loss of brush borders, as well as the formation of casts and luminal debris. Sections were graded as follows: 0: minimal or no lesions; 1: < 25 % of tubules were involved; 2: 25 %-50 % of tubules were involved; 3: > 50 % of tubules were involved (Jablonski et al., 1983[23]). For fluorescent immunocytochemistry, briefly, the paraffin sections were deparaffinized by xylene and then hydrated by immersion into decreasing concentrations of ethanol. At least 30 sections provided and collected onto poly-L-lysine coated cover slips for each independent experiment. After that, for antigen retrieval, slides were pretreated by trypsin solution which can significantly re-open the cross-linked epitopes so that antibodies can quickly stick to target antigens. Non-specific protein binding was blocked by 2 h incubation in 1 % BSA+FBS 10 % in TBS-Tween. Next, the sections were incubated (4 °C) overnight with two primary antibodies including mouse monoclonal antibody against p-NF-κB as inflammatory marker (sc-136548, 1:400; Santa Cruz Biotechnology) and rabbit monoclonal antibody against cleaved caspase-3 as apoptosis marker (9579S, 1:300; Cell Signaling Technology). Thereafter, sections were 6-h (room temperature) incubated with the secondary antibodies (Santa Cruz Biotechnology) including fluorescein isothiocyanate (FITC) conjugated goat anti-mouse IgG (1:500) to detect p-NF-κB, texas red (TR) conjugated goat anti-rabbit IgG (1:500) to detect cleaved caspase-3. All sections were counterstained with 4',6-diamidino-2-phenylindole (DAPI) to visualize the nuclei. Using a fluorescence microscopy images were captured and quantified using the NIH ImageJ analysis program (NIH, Bethesda, MD, USA) (Sanadgol et al., 2017[31]). p-NF-κB and cleaved caspase-3 positive immunoreactivity were measured after background subtraction. Similar threshold level was set for every image, on the dark background and the positive signals were quantified.

### Statistical analysis

Data are expressed as mean ± SEM. Statistical analysis between groups were compared by one-way ANOVA and followed by post hoc Tukey's test. The level of significance was set at p<0.05.

## Results

### Long-term NaHS treatment attenuates the progression of renal dysfunction in 5/6 Nx rats

**Figure 1:**
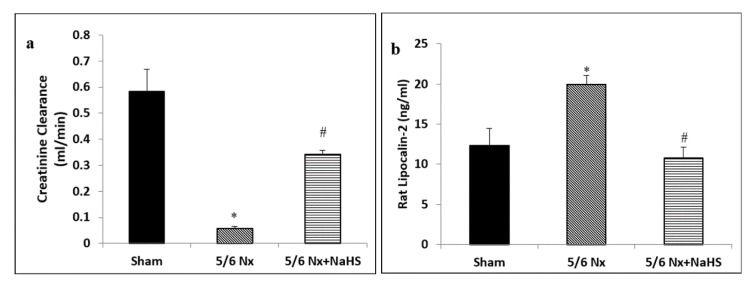
Effect of NaHS on (a) creatinine clearance and (b) urine lipocalin-2 (NGAL) concentration in 5/6 Nx rats. The data are presented as mean ± SEM (n=8). *p<0.05 vs sham group and ^#^p<0.05 vs 5/6 Nx group

Figure 1(Fig. 1) and Table 1(Tab. 1) present the results of treatment with NaHS for 8 weeks on renal function and general characteristics. As shown, creatinine clearance was significantly decreased after 12 weeks in the 5/6 Nx rats as compared with the sham rats (p<0.001). Moreover, other clinical indices such as body weight (p=0.003), blood pressure (p<0.001) and CKD progression, evidenced by NGAL biomarker (p=0.015), were significantly different in sham and 5/6 Nx rats 12 weeks' post-surgery. We noted a significant increase in creatinine clearance (p<0.001) and body weight (p=0.024) after treatment with NaHS. In addition, urine concentration of NGAL (p<0.05) as well as systolic blood pressure p=047) were markedly reduced in 5/6 Nx+ NaHS group as compared to 5/6 Nx subjects.

### Long-term NaHS treatment attenuates renal oxidative stress in 5/6 Nx rats

**Figure 2:**
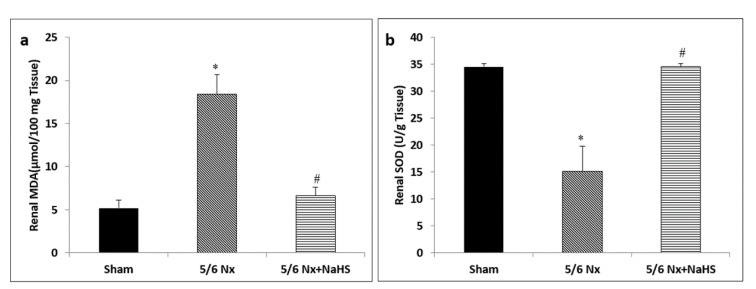
Effect of NaHS on (a) malondialdehyde (MDA) content and (b) superoxide dismutase (SOD) activity in 5/6 Nx rats. The data are presented as mean ± SEM (n=8).*p<0.05 vs sham group and ^#^p<0.05 vs 5/6 Nx group

Five-sixth nephrectomy resulted in significant increase in MDA contents as compared with the sham group (p<0.01). In contrast, NaHS significantly reduced MDA content in 5/6 Nx+ NaHS subjects (p<0.05) as compared with the 5/6 Nx group (Figure 2a(Fig. 2)). Compared with the sham group, 5/6 nephrectomy resulted in significant decrease in SOD activity (p=0.009). The activity of SOD was markedly elevated in the NaHS group (p=0.014) as compared with the 5/6 Nx group (Figure 2b(Fig. 2)).

### Long-term NaHS treatment significantly attenuated histological damage in 5/6 Nx rats

**Figure 3:**
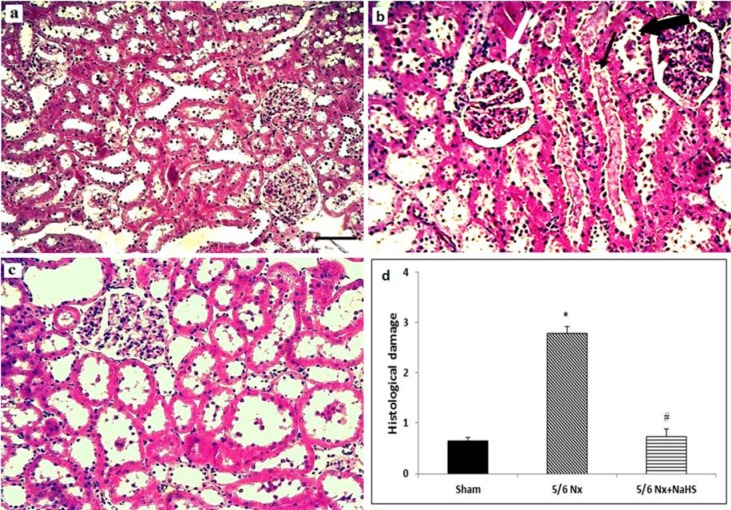
Renal histological changes in the (a) sham, (b) 5/6 nephrectomy (5/6 Nx) and (c) 5/6 Nx+ NaHS groups. The histological changes observed 12 weeks after surgery (a) there was no obvious damage to the glomeruli or tubules in the kidneys of sham group. (b) In the 5/6 Nx group, the most noticeable change was the Bowman's space widening which was present in the glomeruli of the kidneys. There were also extensive tubular dilations which were present in all proximal and distal tubules. Tubular debris was present and leukocytes infiltrations were also frequent in these tissues. (c) In the Nx + NaHS kidneys the degree of Bowman's space widening was lower and also less tubular casts was present comparing to 5/6 Nx group. Magnification ×400; Hematoxylin and Eosin staining. Scale bar is 200 µm. The black thin arrow indicates cast formation, the white arrow shows widening of the Bowman's space. The black thick arrow indicates tubular obstruction. (d) Summary of the histological damage. Histological damage was scored as follows: 0: minimal or no lesions; 1: < 25 % of tubules involved; 2: 25 %-50 % of tubules involved; 3: > 50 % of tubules involved. Data are presented as the mean and range (n=8). *p<0.05 vs sham group and ^#^p<0.05 vs 5/6 Nx group

The histological evaluations showed no obvious signs of damage to the glomeruli or tubules in the kidneys of sham group (Figure 3(Fig. 3)). In the 5/6 Nx group, the most noticeable change was the widening Bowman's space in the glomeruli of the kidneys. There were also extensive tubular dilations present in proximal and distal tubules. Tubular debris was present and leukocytes infiltrations were also frequent in these tissues. In the 5/6 Nx +NaHS kidneys, the degree of Bowman's space widening was lower and less tubular casts were present as compared to 5/6 Nx group. 

### Long-term NaHS treatment significantly attenuated cleaved caspase-3 and p-NF-κB expression in 5/6 Nx rats

**Figure 4:**
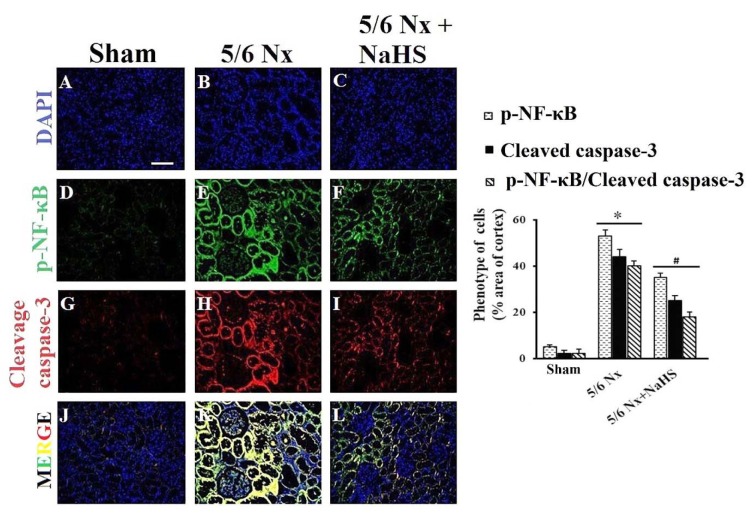
Double stained fluorescence microscopy image of p-NF-κB and cleaved caspase-3 related antigen from paraffin-embedded kidney rat 12 week after sham operation or 5/6 nephrectomy. Five-sixth nephrectomy resulted in overexpression of cleaved caspase-3 and p-NF-κB, whereas treatment with NaHS resulted in milder expression of cleaved caspase-3 and p-NF-κB in comparison with 5/6 Nx subjects. Left side of this figure shows the fluorescence signals obtained with antibodies against p-NF-κB (green) or cleaved caspase-3 (red) and DAPI staining of the DNA (blue). Scale bar is 25 µm. (A-C) nuclei were stained with DAPI (blue). (D) p-NF-κB was stained with secondary antibodies, no p-NF-κB was found in the kidney of rat after sham operation (n = 5-6). (E) p-NF-κB -positive cells (in green) were found to be positive in 5/6 Nx. (F) p-NF-κB -positive cells (in green) were found to be positive in 5/6 Nx+ NaHS rats. (G) No cleaved caspase-3-positive cells were found in the kidney of rat after sham operation (n = 5-6). (H) Cleaved caspase-3-positive cells were found to be positive in 5/6 Nx rats. (I) Cleaved caspase-3-positive cells (in red) were found to be positive in 5/6 Nx+ NaHS rats. (J-L) Double stained fluorescence microcopy image of p-NF-κB and cleaved caspase-3 was obtained. The right side of this figure shows the effects of H_2_S on cleaved caspase-3 and NF-κB expression in kidney tissue 12 weeks after surgery. The data are presented as mean ± SEM (n=8). *p<0.05 vs sham group and^ #^p<0.05 vs 5/6 Nx group

We investigated if the protective effects of NaHS were mediated due to suppression of inflammation (p-NF-κB) and apoptosis (cleaved caspase-3). As shown in Figure 4(Fig. 4), five-sixth nephrectomy resulted in overexpression of cleaved caspase-3 (p=0.021) and p-NF-κB (p<0.001), whereas treatment with NaHS resulted in milder expression of cleaved caspase-3 (p=0.045) and p-NF-κB (p=0.036) in comparison with 5/6 Nx subjects. 

## Discussion

In the present study, we aimed to evaluate the effectiveness of NaHS as a potential candidate for preventing CKD progression. The key findings of our study are: 1) NaHS (donor of H_2_S) reduced blood pressure and increased creatinine clearance in 5/6 nephrectomized rats, which was accompanied by the reduction of NGAL levels, 2) in 5/6 nephrectomized rats, NaHS treatment reduced MDA levels and increased SOD activity. These results were supported by 3) the histological improvements and reduced kidney injury in 5/6 Nx treated rats, and 4) there was a marked decrease in cleaved caspase-3 and p-NF-κB expression after eight weeks of NaHS treatment.

 The presence of hypertension in 5/6 Nx subjects suggests the development of cardiovascular dysfunction after CKD which may increase the morbidity and mortality in these subjects (Askari et al., 2016[4]). To further investigate cardiovascular dysfunction, we separated the hearts of rats at the end of 12 weeks and calculated the ratio of heart/body weight. We found an increased heart/body weight ratio in 5/6 Nx rats in comparison with sham and 5/6 Nx + NaHS groups (data is not shown), suggesting that CKD may have led to cardiac hypertrophy and dysfunction. However, for this notion, functional changes using echocardiography or other appropriate techniques shall be evaluated in future studies.

Progressive increase in the production of reactive species of oxygen (ROS) and subsquent decrease in antioxidants after CKD appears to be the key features for the pathophysiology of CKD (Abboud and Henrich, 2010[1]; Boon et al., 2015[9]). Hence, a significant increase in oxidative stress may stimulate cell hypertrophy and proliferation and inflammatory-cell infiltration (Abboud and Henrich, 2010[1]; Ding et al., 2015[13]) which can lead to increased activity of NF-κB and inflammatory responses. Such inflammatory processes, especially in the presence of ROS may induce cytotoxic effects and cause apoptotic cell death (Esch et al., 2002[15]). In recent years, it has been reported that antioxidants protect the kidney against 5/6 nephrectomy by reducing renal injury in CKD animals through inhibition of proinflammatory factors like NF-κB (Abboud and Henrich, 2010[1]; Ding et al., 2015[13]). Herein, our results showed that tissue concentrations of MDA were significantly higher in 5/6 Nx rats as compared to sham subjects. Furthermore, SOD activity considerably decreased in these subjects. It seems that the overexpression of p-NF-κB and cleaved caspase-3 in CKD animals is in accordance with our observations about hypertrophy and apoptosis in the end stage kidneys which would have contributed to increased serum levels of NGAL and decreased clearance of creatinine (Brisco and Testani, 2014[10]; Khan and Pandey, 2014[25]). Since the use of blood urea nitrogen and serum creatinine due to their low predictive values have some shortcomings as the main biomarkers of CKD, new candidate biomarkers such as neutrophil gelatinase-associated lipocalin (NGAL) have been introduced for early diagnosis of CKD due to its ability to detect tissue damage rather than kidney dysfunction (Fassett et al., 2011[17]; Yim, 2015[39]). NGAL is a secreted glycoprotein and a member of lipocalin family that is characterized as an adipose-derived cytokine involved in the inflammatory and immune responses regulated by NF-κB (Iannetti et al., 2008[22]). It has been shown that exogenous NGAL protects the renal tubular epithelial cells against ischemia/reperfusion injury by reducing apoptosis (Gong et al., 2012[20]) and converting embryonic mesenchymal cells into epithelial cells to form tubules and then the complete nephrons (Khan and Pandey, 2014[25]). However, most of the studies have shown that increased levels of NGAL can predict a degree of kidney dysfunction, i.e. higher expression of NGAL is parallel with kidney injury and alterations in kidney NGAL levels leads to increased uNGAL concentrations (He et al., 2015[21]; Peters et al., 2011[30]; Yim, 2015[39]). In support of this notion, we reported increased uNGAL concentrations in 5/6 Nx rats which may in turn regulate inflammatory states to cause further impairment of kidneys after CKD induction.

In the kidney, the diuretic, natriuretic and kaliuretic effects of H_2_S increase in GFR and inhibit tubular sodium re-absorption (Aminzadeh and Vaziri, 2012[3]). It has been shown that CKD is associated with significant reduction in plasma H_2_S concentration (Aminzadeh and Vaziri, 2012[3]). Since CKD is accompanied by hypoxic conditions, H_2_S may act as an oxygen sensor to restore oxygen balance and cause increased medullary blood flow and GFR (Beltowski, 2010[7]). The present study established a clear link between NaHS treatment in 5/6 Nx rats and increased clearance of creatinine, suggesting that NaHS may have improved creatinine clearance by enhancing blood flow to the kidneys. Moreover, our observation about the reduction in uNGAL after NaHS treatment suggests the ability of H_2_S to alleviate kidney damage in CKD. Similarly, recent work of Sen et al. (2009[32]) indicated that H_2_S decreases renal damage in a rat model of hyperhomocysteinemia-associated chronic renal failure. We also assessed the impact of H_2_S on histological parameters after 5/6 nephrectomy. Histological analysis revealed a substantial decrease in glomerular hypertrophy and Bowman's space widening as well as a decrease in leukocytes infiltration within nephrectomized kidneys. Additionally, we showed that H_2_S potently reduces renal apoptosis detected by decreased activity of cleaved caspase-3. Inhibition of cleaved caspase-3 activity is one possible mechanism by which H_2_S attenuates the extent of apoptosis and thereby confines the extent of CKD progression (Ford et al. 2013[19]). Due to the antioxidant functions of H_2_S, it has been proposed that in *in vivo* and *in vitro* models, the long-term treatment with H_2_S significantly protects the kidney from renal injury by limiting the extent of oxidative damages (Lin et al., 2016[27]). In the present study, we examined the effects of NaHS for 8 weeks on 5/6 Nx rats and found that H_2_S treatment attenuated MDA levels and NF-κB activity, and increased SOD activity at week 12 after induction of 5/6 nephrectomy, suggesting its long-term renoprotective effects against CKD. Therefore, antioxidant and anti-inflammatory actions of H_2_S may play critical roles in prevention of 5/6 Nx-induced renal oxidative damage. Furthermore, regarding our observation about uNGAL reduction after NaHS administration, it is also plausible that H_2_S has an inhibitory effect on p-NF-κB. Previous evidences have indicated that NF-κB mediates inflammatory processes (Baeuerle and Baltimore ,1996[6]; Chen et al., 2009[11]). In non-unstimulated states, NF-κB dimers are maintained in the cytosol in collaboration with the inhibitor of nuclear factor-κB (IκB). Robust elevation of pro-inflammatory cytokines such as tumor necrosis factor-α (TNF-α), as a consequence of hypoxic cell death of glomeruli after CKD induces an inflammatory response which is responsible for IκB release from NF-κB. Activation and localization of p-NF-κB in the nucleus regulates the expression of proinflammatory cytokines and inflammatory mediators, such as NGAL (Avci Cicek et al., 2016[5]; Lian et al., 2010[26]). Our results also suggest that NGAL levels are related to p-NF-κB changes. Considering the potent anti-oxidant and anti-inflammatory properties of H_2_S (Aminzadeh and Vaziri, 2012[3]), it seems that NaHS may, in part, contribute to confine CKD progression by NF-κB and cleaved caspase-3 down-regulation after 5/6 nephrectomy. 

Recently, Wu et al. (2017[38]) investigated the effect of hydrogen sulfide using a rat model of adenine-induced CRF. Various methods, are available for inducing the model of chronic kidney disease and none of them can ever duplicate the original condition in a human kidney. Among these ways to induce experimental renal failure is via the administration of nephrotoxic chemical agents including adenine and uranyl nitrate administration. The disadvantage is obvious biologic or pathologic effects of these chemical agents on the lymphoid and other organ systems would be difficult to control and monitor (Chow et al., 2003[12]; Zhang and Kompa, 2014[40]). In our study using 5/6 nephrectomy model has remained the developed prototype which closely mimics human CKD and is a useful model for studying drug responses in CKD. In contrast to what was observed in 5/6 nephrectomy, adenine diet failed to induce kidney failure in rabbits as is evident in functional indices in a recent study (Florens et al., 2017[18]). In the current study, we investigated the effects of H_2_S on CKD induced by 5/6 Nx in different dose, administration method and longer time in rats. Moreover, we evaluated NGAL level which is suggestive of renal injury (Bolignano et al., 2009[8]). 

## Conclusions

Taken together, these findings indicate that long-term treatment by NaHS inhibits ROS injury and renal damages through inhibition of apoptosis and inflammation. Therefore, NaHS can be considered as complementary therapeutic agent in the protection against kidney damage in chronic kidney disease.

## Acknowledgements

This research was supported by a grant from Tehran University of Medical Sciences (no=28769).

## Disclosure

The authors declare that they have no conflict of interest.

## Figures and Tables

**Table 1:**
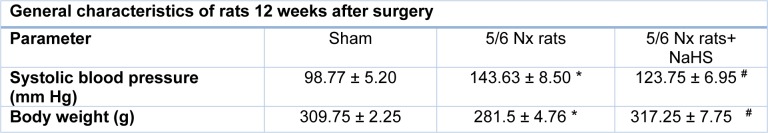
Effect of NaHS on systolic blood pressure and body weight in 5/6 Nx rats. The data are presented as mean ± SEM (n=8). *p < 0.05 vs sham group. ^#^p < 0.05 vs 5/6 Nx group
